# DJ-1 controls T cell differentiation and osteoclastogenesis in rheumatoid arthritis

**DOI:** 10.1038/s41598-022-16285-1

**Published:** 2022-07-27

**Authors:** Hong Ki Min, Se Hee Kim, Ji-Yeon Lee, Sang-Heon Lee, Hae-Rim Kim

**Affiliations:** 1 Division of Rheumatology, Department of Internal Medicine, Konkuk University Medical Center, Seoul, 05030 Republic of Korea; 2 The Rheumatism Research Center, Research Institute of Medical Science, Konkuk University School of Medicine, Seoul, 05030 Republic of Korea; 3 Division of Rheumatology, Department of Internal Medicine, Research Institute of Medical Science, Konkuk University Medical Center, Konkuk University School of Medicine, 120-1, Neungdong-ro, Gwangjin-gu, Seoul, 05030 Republic of Korea

**Keywords:** Immunology, Rheumatology

## Abstract

Herein, we investigated the effect of DJ-1 on helper T cell differentiation, fibroblast-like synoviocyte (FLS) activation, and osteoclastogenesis in rheumatoid arthritis (RA). Serum and synovial fluid (SF) of RA and osteoarthritis (OA) patients were collected, and DJ-1 and H_2_O_2_ levels were investigated. CD4^+^ cells from peripheral blood mononuclear cells (PBMCs) were cultured under type 17 helper T cell (Th17) polarization conditions, and CD4^+^ T cell differentiation, pro-inflammatory cytokine levels, and soluble receptor activator of nuclear factor kappa-Β ligand (RANKL) were assessed. RA-FLSs were stimulated with 50 μM H_2_O_2_, and DJ-1 (10, 50, 100 ng/mL) to evaluate MMP-9, VEGF, TNF-α, and sRANKL production, while RANKL^+^ FLSs were assessed using flow cytometry. Monocytes were cultured with RANKL or IL-17A with or without DJ-1 and H_2_O_2_-pretreated RA-FLS, and tartrate-resistant acid phosphatase (TRAP) staining and RT-qPCR of osteoclast-related genes were performed. The levels of DJ-1 and H_2_O_2_ in serum and SF of RA patients were higher than those of OA patients. Under Th17-polarizing conditions, CD4^+^RANKL^+^ and CD4^+^CCR4^+^CCR6^+^CXCR3^-^ T cells decreased, whereas CD4^+^CD25^high^Foxp3^+^ T cell increased after DJ-1 administration. Additionally, IL-17A, TNF-α, and sRANKL levels decreased in DJ-1-treated groups. DJ-1 lowered MMP-9, VEGF, TNF-α, and sRANKL levels, and RANKL^+^ FLS in ROS-stimulated RA-FLS. Both RANKL and IL-17A stimulated osteoclast differentiation, DJ-1 decreased TRAP^+^ cell count, and the expression levels of *TRAP, ATP6v0d2, NFATc1*, and *CTSK*. These findings were also observed in in vitro osteoclastogenesis with DJ-1 pretreated RA-FLS. As DJ-1 regulates Th17/Treg imbalance, pro-inflammatory cytokine production, RA-FLS activation, and osteoclastogenesis, it holds potential for RA therapy.

## Introduction

Rheumatoid arthritis (RA) is an autoimmune-mediated systemic arthritis that affects 0.5–1% of the general population^1^. The pathogenesis of RA is complicated, involving both immunological and environmental factors^2^. Smoking is the best-known environmental risk factor for RA^3^, which has been shown to enhance citrullination in a mouse model of RA^4^. *HLA-DRB1*, encoding the shared epitope, has been identified as a susceptibility gene for RA occurrence^5^, and several potential susceptibility genes have been identified in genome-wide association studies^6^. Post-translational modifications, such as citrullination, carbamylation, glycation, and methylation, modify self-tolerable proteins into auto-antigens and induce autoantibodies such as anti-citrullinated peptide antibodies (ACPA)^7,8^. These complex pathogeneses result in pannus formation and consequent joint destruction that are the hallmarks of RA^1^. Aggravated synovitis, which eventually forms a pannus, is the cornerstone pathologic change of the joint in RA. In the pannus, reactive oxygen species (ROS) induce pro-inflammatory cytokine production, pathologic cell recruitment, and osteoclastogenesis^9–12^. Furthermore, ROS-bound type II collagen has shown higher reactivity in early ACPA-positive RA patients than in ACPA-positive arthralgia patients^13^. The serum levels of ROS positively correlate with RA disease activity^14^. Pro-inflammatory cytokine-stimulated RA fibroblast-like synoviocytes (FLS) show enhanced migration and invasion, and increased ROS levels in these processes^15^. Furthermore, oxidative stress can activate RA-FLSs^16^. Vigorous angiogenesis is a characteristic change of the pannus, mediated by vascular growth factors such as vascular endothelial growth factor (VEGF)^17^. ROS promotes angiogenesis by upregulating the expression of genes associated with angiogenesis^18^.

In RA synovitis, various immune and periarticular cells, such as synoviocytes and osteoclasts, are involved in the progression of RA^1,2^. In RA, type 17 helper T cell counts (Th17) are increased and play a pathologic role, whereas regulatory T cells (Tregs) is decreased in number, and it suppress the autoimmune-mediated process of RA^19,20^. The main effector cells of joint destruction in RA are the FLSs and osteoclasts. Osteoclast differentiation and consequent osteoporosis and joint destruction are more prominent in patients with RA than in healthy controls^21,22^. Osteoclast differentiation is fundamentally induced by receptor activator of NF-κB (RANK)-RANK ligand (RANKL) binding. In RA, RANKL expression of helper T cells and FLSs is upregulated^23,24^; furthermore, some pro-inflammatory cytokines, such as interleukin (IL)-17A, induce osteoclastogenesis^25^. Therefore, proper management of Th17/Treg imbalance, enhanced osteoclastogenesis, and activated FLS are treatment strategies for RA.

DJ-1 a protein comprised of 189 amino acids, is encoded by the *PARK7* gene^26^. It was first identified as a protective protein in Parkinson’s disease^27^, in which it primarily acts as a ROS scavenger and involved in redox reaction^28^. DJ-1 is expressed in various cells of the central nervous system including astrocytes, microglia, and oligodendrocytes^29^. DJ-1 not only has a neuroprotective role, but also suppresses osteoclast differentiation by scavenging ROS^30^ and maintaining metabolic homeostasis of skeletal muscle by modulating the mitochondrial function^31^. Moreover, the extracellular administration of DJ-1 induces angiogenesis and osteogenesis via FGF receptor-1 signaling which implies that DJ-1 not only exert intracellular effect but the extracellular administration of DJ-1 may also have roles in various cells^32^. Furthermore, DJ-1 deficiency-induced Tregs show reduced proliferative potential^33^. Tregs act as self-tolerance inducers and are negatively associated with RA activity^34^. In a study of collagen-induced RA mice, DJ-1-deficient mice showed higher arthritis index and incidence rate than wild-type mice^30^. The aforementioned actions of DJ-1 suggest a potential therapeutic role for DJ-1 in RA progression.

In the present study, we aimed to evaluate the role of DJ-1 in Th cell differentiation. Furthermore, the effect of DJ-1 on RA patients with FLS was assessed. Finally, osteoclastogenesis induced by pro-inflammatory cytokines (IL-17A), RANKL, and RA-FLS was evaluated under various doses of DJ-1.

## Materials and methods

### Patients

Serum samples were obtained from patients with RA (n = 57) and those with osteoarthritis (OA, n = 30); synovial fluid (SF) samples from patients with RA (n = 30) and those with OA (n = 30) were also collected. The inclusion criteria for patients with RA were as follows: (1) fulfillment of the 2010 classification criteria for RA^35^, and (2) age over 18 years. Peripheral blood was obtained from healthy controls (n = 10). The present study was conducted in accordance with the Declaration of Helsinki and Good Clinical Practice guidelines. Informed consent was obtained from all the participants prior to enrolment. The experimental protocol was approved by the Konkuk University Medical Center Human Research Ethics Committee (KUMC 2021-06-007).

### Enzyme-linked immunosorbent assay (ELISA) of DJ-1 and ROS measurement in serum and SF

Briefly, a 96-well plate (Eppendorf, Hamburg, Germany) was coated with monoclonal antibodies against Park7/DJ-1, tumor necrosis factor (TNF)-α, IL-17A, RANKL, IL-6, matrix metalloproteinase (MMP)-9, VEGF (all from R&D Systems, Minneapolis, MN, USA), and IL-1β (PeproTech, Cranbury, NJ, USA) at a concentration of 400 ng/mL and incubated at 4 °C overnight. After blocking with phosphate-buffered saline (PBS)/1% bovine serum albumin /0.05% Tween 20 for 2 h at room temperature (22–25 °C), the test samples and recombinant Park7/DJ-1, TNF-α, IL-17A, RANKL, IL-6, MMP-9, VEGF (all from R&D Systems), and IL-1β (PeproTech) as standards were added to the 96-well plate and incubated at room temperature for another 2 h. The plates were washed four times with PBS and Tween 20 and then incubated with 300 ng/mL biotinylated mouse monoclonal antibodies against Park7/DJ-1, TNF-α, IL-17A, RANKL, IL-6, MMP-9, VEGF (all from R&D Systems), and IL-1β (PeproTech) for 2 h at room temperature. After washing, the streptavidin–alkaline phosphate–horseradish peroxidase conjugate (Sigma, St Louis, MA, USA) was added to the wells for 30 min, followed by another wash and incubation with 1 mg/mL *p*-nitrophenyl phosphate (Sigma) dissolved in diethanolamine (Sigma) to develop the color reaction. The reaction was stopped by the addition of 1 M NaOH, and the optical density of each well was measured at 405 nm. The lower limit of Park7/DJ-1 was 10 pg/mL. Recombinant human DJ-1 diluted in the culture medium was used as a calibration standard, ranging from 62.5 to 4000 pg/mL. A standard curve was drawn by plotting the optical density against the log of the concentration of the recombinant cytokines, and the curve was used to determine the Park7/DJ-1 concentrations in the test samples.

Similarly, ROS levels were measured using the OxiSelect in vitro ROS/RNS Assay Kit (Cell Biolabs, San Diego, CA, USA). The test sample and the H_2_O_2_ standard (Cell Biolabs) were loaded onto a shaded 96-well plate (SPL, Gyeonggi-do, Republic of Korea) under dark conditions, and 1 × catalyst solution was added to each well, followed by incutation at room temperature for 5 min. Then, dichlorodihydrofluorescein (DCFH) solution was added to the wells and incubated for 30 min at room temperature. ROS fluorescence (excitation, 485 nm; emission, 535 nm) was analyzed using a GeminiEM fluorescence microplate reader (Molecular Devices, Sunnyvale, CA, USA). H_2_O_2_ diluted in PBS was used as a calibration standard, ranging from 0.039 to 20 µM.

### In vitro culture of CD4^+^ T cells under Th17-polarizing conditions with DJ-1

Peripheral blood mononuclear cells (PBMCs) were extracted from the heparinized peripheral blood of healthy controls (n = 5) using the standard Ficoll-Paque density gradient method (GE Healthcare Biosciences, Uppsala, Sweden). CD4^+^ T cells were purified using EasySep Human CD4^+^ T Cell Isolation Kit (Cat: 17952, STEMCELL Technologies, Vancouver, Canada). The cell culture plate was pre-incubated with an anti-CD3 antibody (1 μg/mL) for 1 h at 37 °C. CD4^+^ cells (1 × 10^6^) were plated in 24-well plates (Nunc) and incubated with anti- interferon-gamma (IFN-γ, 2 μg/mL) and anti-IL-4 (2 μg/mL) antibodies for 4 h. Anti-CD28 antibody (1 μg/mL), IL-1β (20 ng/mL), IL-6 (20 ng/mL), IL-23 (20 ng/mL; R&D Systems), and DJ-1 (0, 10, 50, and 100 ng/mL; Mybiosource, San Diego, CA, USA) were added and the cells were cultured for 72 h.

### *FLS stimulation with H*_*2*_*O*_*2*_

FLSs were isolated with enzymatic digestion of synovial tissues obtained from RA patients undergoing synovectomy surgery, as previously described^36^. RA-FLSs (1 × 10^6^) were plated in 24-well plates (Nunc) and stabilized for 24 h. Subsequently, RA-FLS was stimulated with or without 50 μM H_2_O_2_ (Sigma), 50 μM H_2_O_2_ (R&D Systems) + 10 ng/mL DJ-1, 50 μM H_2_O_2_ + 50 ng/mL DJ-1, and 50 μM H_2_O_2_ + 100 ng/mL DJ-1 for 72 h.

### Flow cytometry

To quantify IFN-γ^+^, CCR4^+^CCR6^+^CXCR3^−^, RANKL^+^, and CD25^+^ forkhead box P3 (FOXP3)^+^ cells in CD4^+^ T cells, CD4^+^ cells were cultured under Th17 polarization conditions with various doses of DJ-1. Subsequently, these cells were immunostained using a PerCP-conjugated anti-CD4 antibody (BD Biosciences, San Jose, CA, USA) and then fixed and permeabilized using a Cytofix/Cytoperm Plus kit (BD Biosciences). Following the manufacturer’s instructions, the cells were stained with fluorescein isothiocyanate-conjugated anti-IFN-γ (BD Biosciences), phycoerythrin-conjugated anti-CD194 (CCR4, BioLegend), fluorescein isothiocyanate-conjugated anti-CD196 (CCR6, BioLegend), allophycocyanin-conjugated anti-CD183 (CXCR3, BioLegend), phycoerythrin-conjugated anti-RANKL (eBiosciences), allophycocyanin-conjugated anti-CD25 (BD Biosciences), and phycoerythrin-conjugated anti-FOXP3 (BioLegend, San Diego, CA, USA) antibodies. To quantify RANKL^+^ RA-FLSs, RA-FLSs were stained with phycoerythrin-conjugated anti-RANKL (eBiosciences). All cells were detected using a FACSCalibur flow cytometer (BD Pharmingen, Franklin Lakes, NJ, USA).

### Osteoclast formation

CD14^+^ monocytes were prepared from the PBMCs of healthy donors (n = 6) using microbeads (Miltenyi Biotec, Auburn, CA, USA). Human CD14^+^ monocytes were seeded in 24-well plates at a density of 1 × 10^6^ cells/well in 1 mL of α-minimum essential medium supplemented with 10% heat-inactivated fetal bovine serum and 25 ng/mL recombinant human macrophage colony-stimulating factor (rhM-CSF, R&D system) for 3 days. Monocytes were cultured with RANKL (20 ng/mL), M-CSF (25 ng/mL), IL-17A (50 ng/mL), and M-CSF (25 ng/mL) for 10–14 days. DJ-1 was added at 0, 10, 50, and 100 ng/mL to evaluate its effect on osteoclastogenesis. The culture medium was changed every 3 days. After 10–14 days, tartrate-resistant acid phosphatase (TRAP)^+^ cells were identified using the TRAP staining kit (Cosmo Bio, Tokyo, Japan), as previously described^37^. A bone resorption assay kit (Cosmo Bio) was used to evaluate the effects of DJ-1 on the functional abilities of osteoclasts. CD14^+^ monocytes (n = 5) were plated on a bone plate under M-CSF (25 ng/mL) and RANKL (20 ng/mL) stimulation with various concentrations of DJ-1 (0, 10, 50, and 100 ng/mL) and then cultured for 10–14 days. The resorption pit area (%) was calculated by using ImageJ software.

### Co-culture of RA-FLS and osteoclasts

RA-FLSs were pre-stimulated with H_2_O_2_ at 50 μM with (10, 50, or 100 ng/mL) or without DJ-1 for 72 h. Human CD14^+^ monocytes (1 × 10^6^ cells/well) were seeded in 24-well plates with α-minimal essential medium and 10% heat-inactivated FBS in the presence of 25 ng/mL rhM-CSF for 72 h. Pre-stimulated RA-FLSs (2 × 10^3^ cells/well) were added to the osteoclast culture plate. As a control, non-stimulated RA-FLS-added conditions were used as controls. The medium was supplemented with RANKL (40 ng/mL) and rhM-CSF (30 ng/mL) when RA-FLS was added. The culture medium was changed every 3 days. On days 10–14, TRAP^+^ cells were identified using a TRAP staining kit (Cosmo Bio)^37^.

### RNA preparation and quantification of gene expression levels using real-time quantitative polymerase chain reaction (RT-qPCR)

Total RNA was extracted using an easy-spin Total RNA Extraction Kit (Intron Biotechnology, Seongnam, Republic of Korea), according to the manufacturer’s instructions. RNA samples were then quantified, aliquoted, and stored at − 80 °C until analysis. Total RNA (500 ng) was reverse-transcribed to cDNA using an AccuPower CycleScript RT PreMix cDNA synthesis kit (Bioneer, Daejeon, Republic of Korea) according to the manufacturer’s instructions. qPCR was performed in a total volume of 20 μL containing 7.2 μL of PCR-grade distilled water, 0.4 μL of forward and reverse primers, and 10 μL of the SYBR Green I Master mix (Roche Diagnostics, Mannheim, Germany). The PCR conditions were as follows: 95 °C for 10 min, followed by 40 cycles of 95 °C for 15 s, 59 °C for 15 s, and 72 °C for 15 s. All primers were synthesized by Bioneer Corp. (Daejeon, Republic of Korea). Relative mRNA expression levels were normalized to *ACTB* mRNA levels.

### Statistical analysis

All data are expressed as the mean ± standard error of the mean. Statistical analysis was performed using one-way analysis of variance followed by Bonferroni’s post-hoc multiple comparison test. Statistical significance was set at *P* < 0.05. All statistical analyses were performed using Prism 9.0 (GraphPad Software Inc., San Diego, CA, USA).

## Results

### ***Serum and SF levels of DJ-1 and H***_***2***_***O***_***2***_*** in patients with RA***

**Figure 1:**
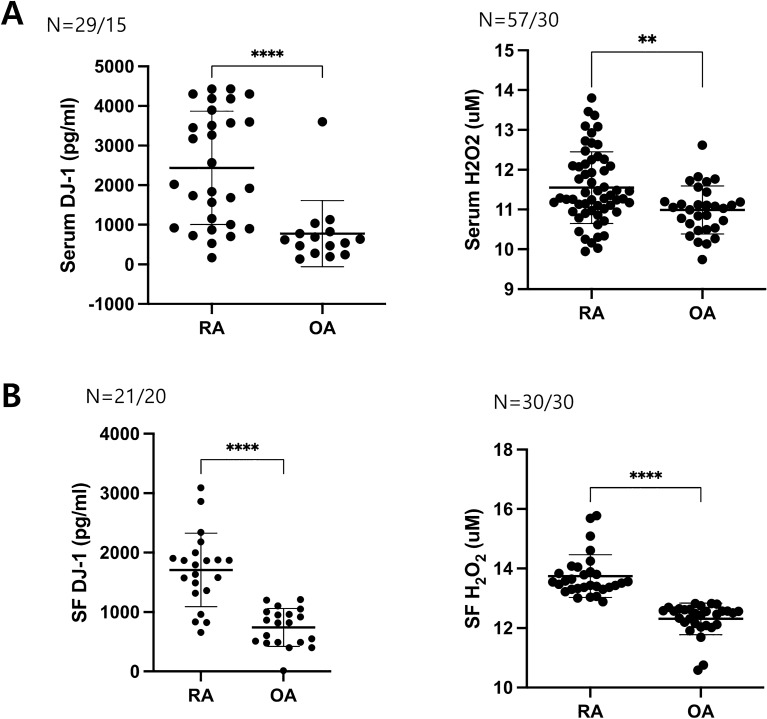
Serum and synovial fluid levels of DJ-1 and H_2_O_2_ in patients with rheumatoid arthritis (RA) and osteoarthritis (OA). (**A**) Serum levels of DJ-1 (RA = 29, OA = 15) and H_2_O_2_ (RA = 57, OA = 30) measured using ELISA or ROS Assay Kit in RA and OA patients. (**B**) Synovial fluid levels of DJ-1 (RA = 21, OA = 20) and H_2_O_2_ (RA = 30, OA = 30) measured using ELISA or ROS Assay Kit in RA and OA patients. **P < 0.01, ****P < 0.0001.

The serum levels of DJ-1 and H_2_O_2_ were significantly higher in patients with RA than in those with OA (Fig. 1A). Higher levels of DJ-1 and H_2_O_2_ in RA patients were also observed in the SF (Fig. 1B). The serum levels of H_2_O_2_ were higher in patients with active RA (DAS28-ESR > 3.2) than in those with inactive disease (DAS28-ESR ≤ 3.2), whereas DJ-1 levels were higher in patients with inactive RA (data not shown).

### Effects of DJ-1 on Th differentiation and pro-inflammatory cytokines and soluble RANKL production under Th17-polarizing condition

**Figure 2:**
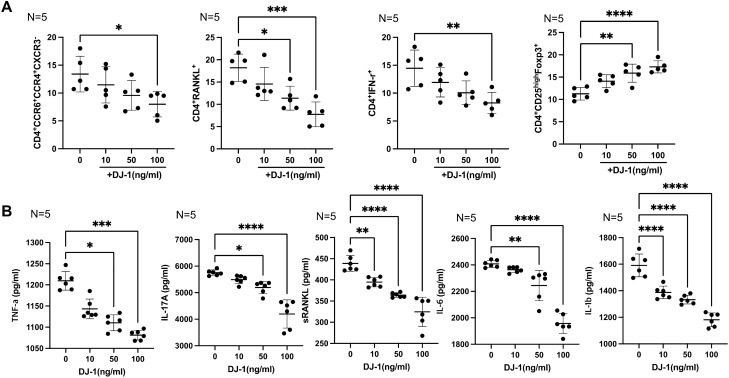
CD4^+^ T cell differentiation of peripheral blood mononuclear cells (PBMCs) determined using flow cytometry, and levels of pro-inflammatory cytokines and soluble RANKL in culture media with Th17-polarizing conditions. PBMCs from healthy controls (n = 5) were cultured under Th17 conditions (IL-1β, IL-6, IL-23 added media) with DJ-1 (10, 50, 100 ng/mL). (**A**) CD4^+^ T cell (CD4^+^ RANKL^+^ T cells, CD4^+^ CCR4^+^ CCR6^+^ CXCR3^−^ T cells, CD4^+^ CD25^high^ Foxp3^+^ T cells, and CD4^+^IFN-γ^+^ T cells).differentiation assessed using flow cytometry. (**B**) Levels of TNF-α, IL-17A, soluble RANKL, IL-6, IL-1β in culture media determined using ELISA. *P < 0.05, **P < 0.01, ***P < 0.001, ****P < 0.0001.

In vitro CD4^+^ cell culture was performed under Th17-polarizing conditions to mimic RA conditions, and the CD4^+^ cell populations were analyzed using flow cytometry. The gating strategy is shown in Supplementary Fig. S1. Flow cytometry analysis revealed that DJ-1 decreased the CD4^+^CCR6^+^CCR4^+^CXCR3^−^ T cell (Th17) population in a dose-dependent manner. CD4^+^IFN-γ^+^ T cells (Th1) and CD4^+^RANKL^+^ T cell numbers were suppressed following DJ-1 administration. CD4^+^CD25^high^Foxp3^+^ T cell differentiation was significantly increased by DJ-1 treatment in a dose-dependent manner (Fig. 2A). DJ-1 also suppressed the production of tumor necrosis factor (TNF)-α, IL-17A, and sRANKL in culture media (Fig. 2B).

### ***FLS activation by H***_***2***_***O***_***2***_*** stimulation and inversion by DJ-1 treatment***

**Figure 3:**
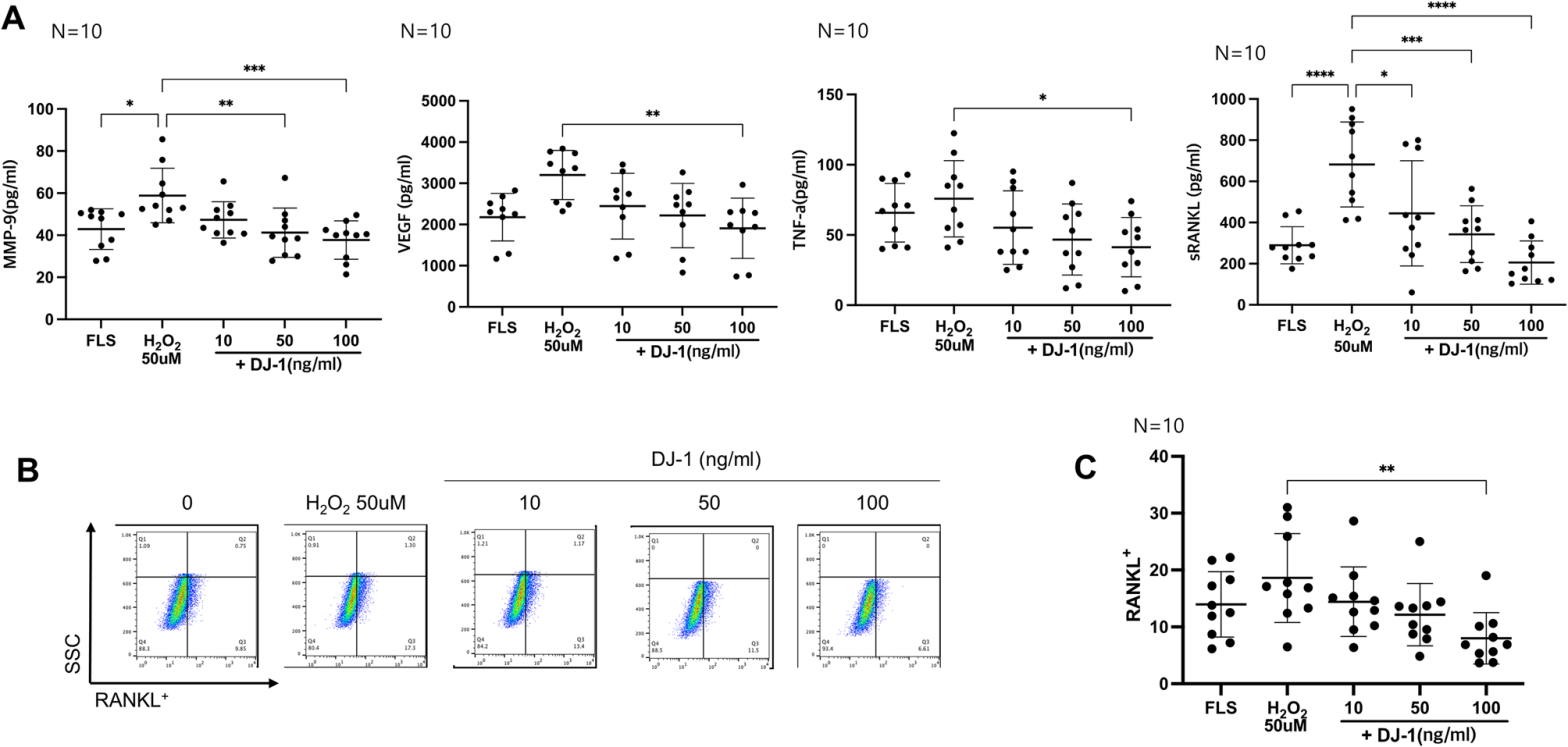
H_2_O_2_-stimulated RA-FLS activation measured using ELISA and flow cytometry. FLS from RA patients (n = 10) were cultured with 50 μM H_2_O_2_ with or without DJ-1 (10, 50, and 100 ng/mL) (**A**) ELISA of the levels of MMP-9, VEGF, TNF-α, and soluble RANKL in culture media. (**B**) The flow cytometry gating strategy for RANKL^+^ FLSs (**C**) RANKL^+^ FLS differentiation measured using flow cytometry. *P < 0.05, **P < 0.01, ****P < 0.0001.

The demographic, laboratory, and medication data of RA patients were summarized in Supplementary Table 1. When RA-FLSs were cultured with H_2_O_2,_ they produced more MMP-9 and sRANKL than the RA-FLSs cultured without H_2_O_2_. DJ-1 administration decreased the H_2_O_2_-induced production of MMP-9 and RANKL in a dose-dependent manner. Further, the levels of VEGF and TNF-α significantly decreased after DJ-1 100 ng/mL administration (Fig. 3A). The gating strategy for this flow cytometric analysis is shown in Fig. 3B. RANKL^+^ FLS were also suppressed in the DJ-1 100 ng/mL administration group (Fig. 3C).

### DJ-1 suppresses osteoclast differentiation under RANKL and IL-17A induced osteoclastogenesis

**Figure 4:**
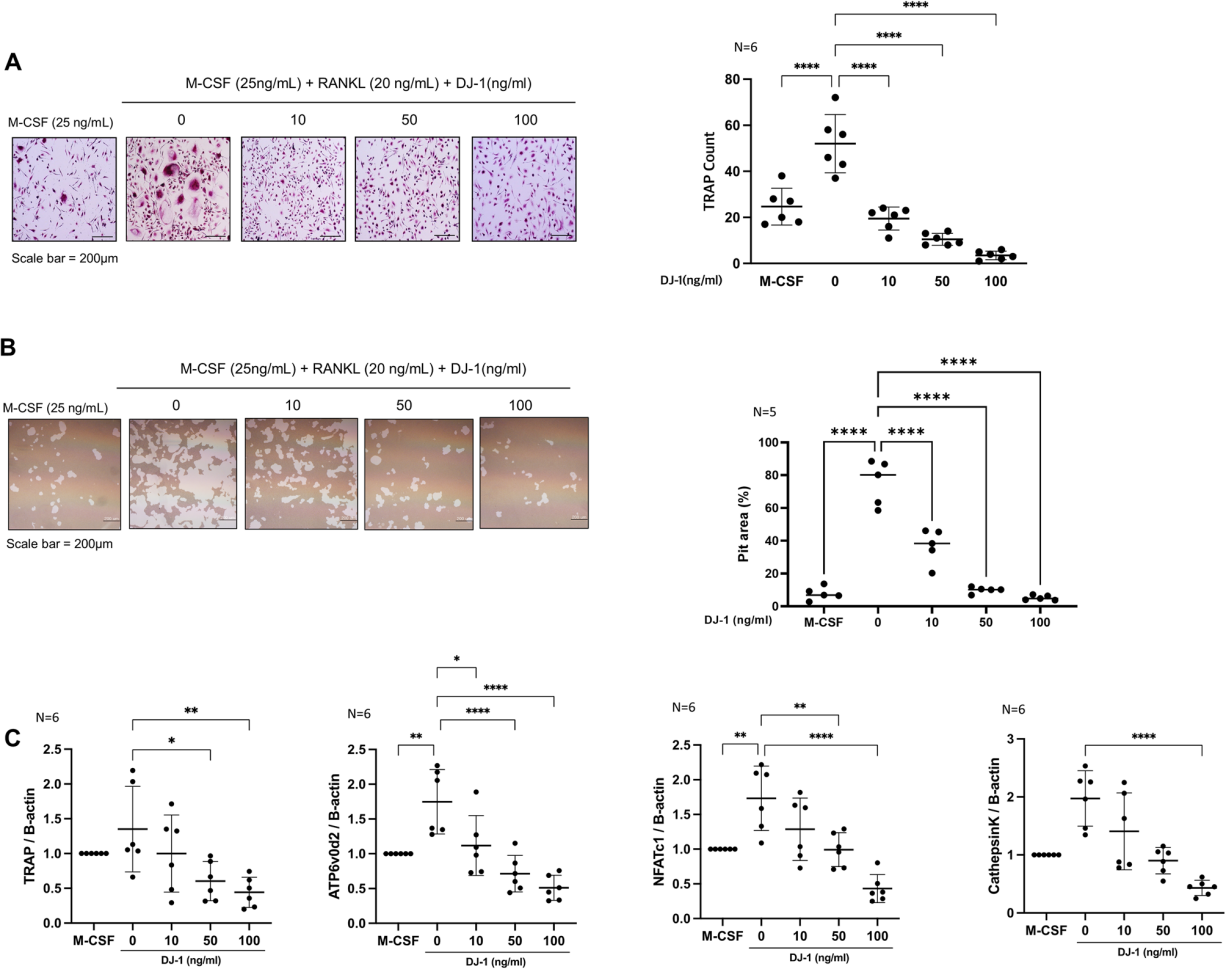
Suppressive role of DJ-1 on osteoclastogenesis under RANKL stimulation. CD14^+^ monocytes from HC (n = 6) were cultured with RANKL (30 ng/mL) + M-CSF (25 ng/mL) without or with DJ-1 (10, 50, 100 ng/mL). (**A**) Number of TRAP^+^ multinucleated cells. (**B**) Calculation of the resorptive pit area. (**C**) Gene expression of *TRAP, OC-sTAMP, ATP6v0d2, NFATc1,* and *CTSK* from osteoclasts measured using RT-qPCR. Data were normalized to *ACTB* expression and are reported in relative expression units. *P < 0.05, **P < 0.01, ***P < 0.001, ****P < 0.0001. Scale bar = 200 μm.

**Figure 5:**
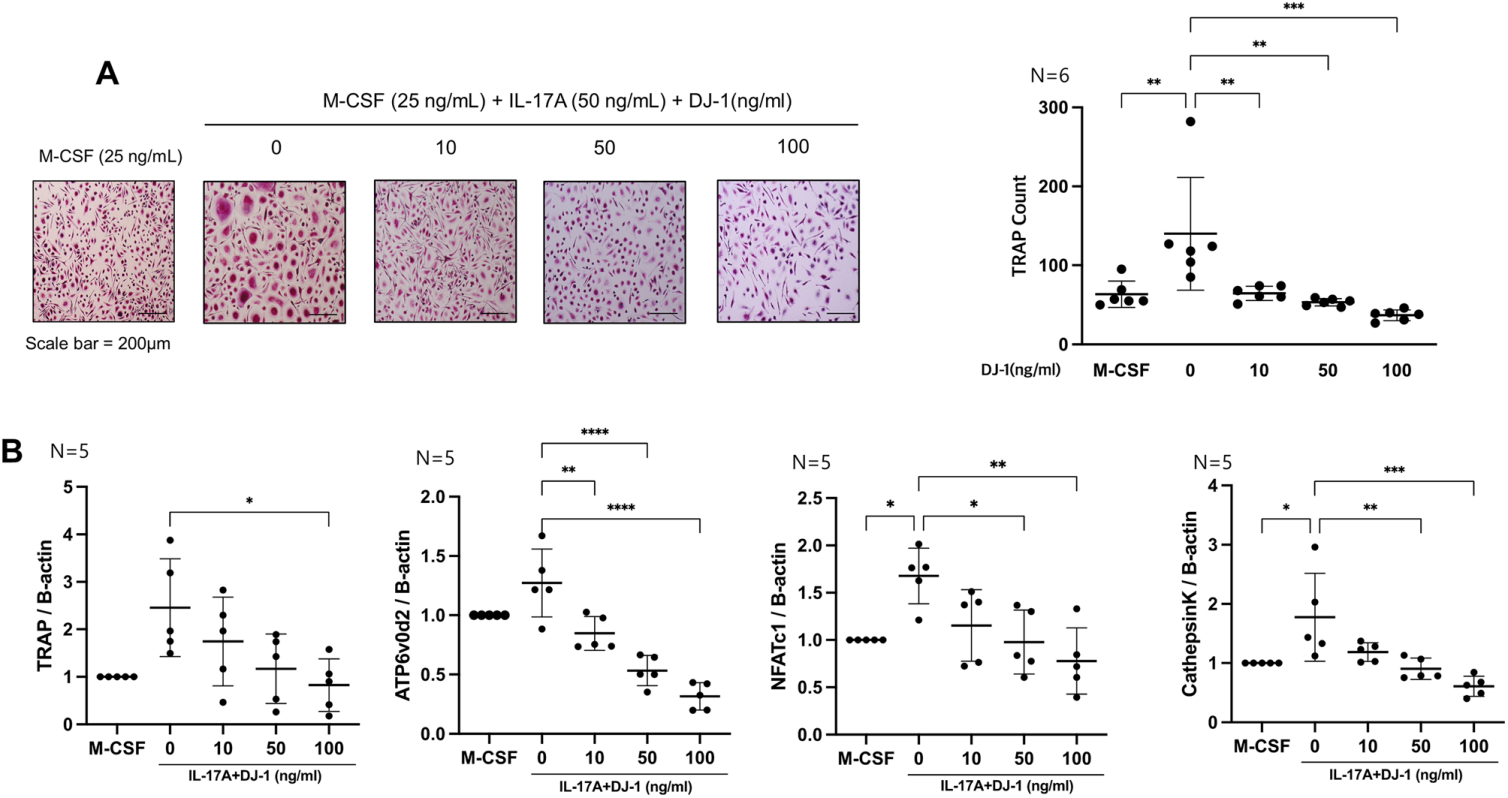
Suppressive role of DJ-1 on osteoclastogenesis under IL-17A stimulation. CD14^+^ monocytes from HC (n = 6) were cultured with IL-17A (50 ng/mL) + M-CSF (25 ng/mL) without or with DJ-1 (10, 50, 100 ng/mL). (**A**) Number of TRAP^+^ multinucleated cells. (**B**) Gene expression of *TRAP, OC-sTAMP, ATP6v0d2, NFATc1,* and *CTSK* osteoclasts measured using RT-qPCR. Data were normalized to *ACTB* expression and are reported in relative expression units. *P < 0.05, **P < 0.01, ***P < 0.001, ****P < 0.0001. Scale bar = 200 μm.

TRAP^+^ multinucleated cells were counted to evaluate osteoclast differentiation. DJ-1 dose-dependently decreased the TRAP^+^ cell counts in RANKL-stimulated cells (Fig. 4A). In addition, the resorptive pit area (%) was decreased by DJ-1 addition (Fig. 4B). In RT-qPCR, osteoclast-related gene (*TRAP, ATP6v0d2, NAFTc1,* and *CTSK*) levels were suppressed in the DJ-1 treated group in a dose-dependent manner relative to RANKL stimulation without DJ-1 (Fig. 4C). The TRAP^+^ cell count was also decreased in the DJ-1 treatment group under IL-17A stimulated condition (Fig. 5A). The expression levels of osteoclast-related genes (*TRAP, ATP6v0d2, NAFTc1,* and *CTSK*) were significantly suppressed in the DJ-1 addition groups (Fig. 5B).

### DJ-1-stimulated RA-FLS reduce osteoclastogenesis

**Figure 6:**
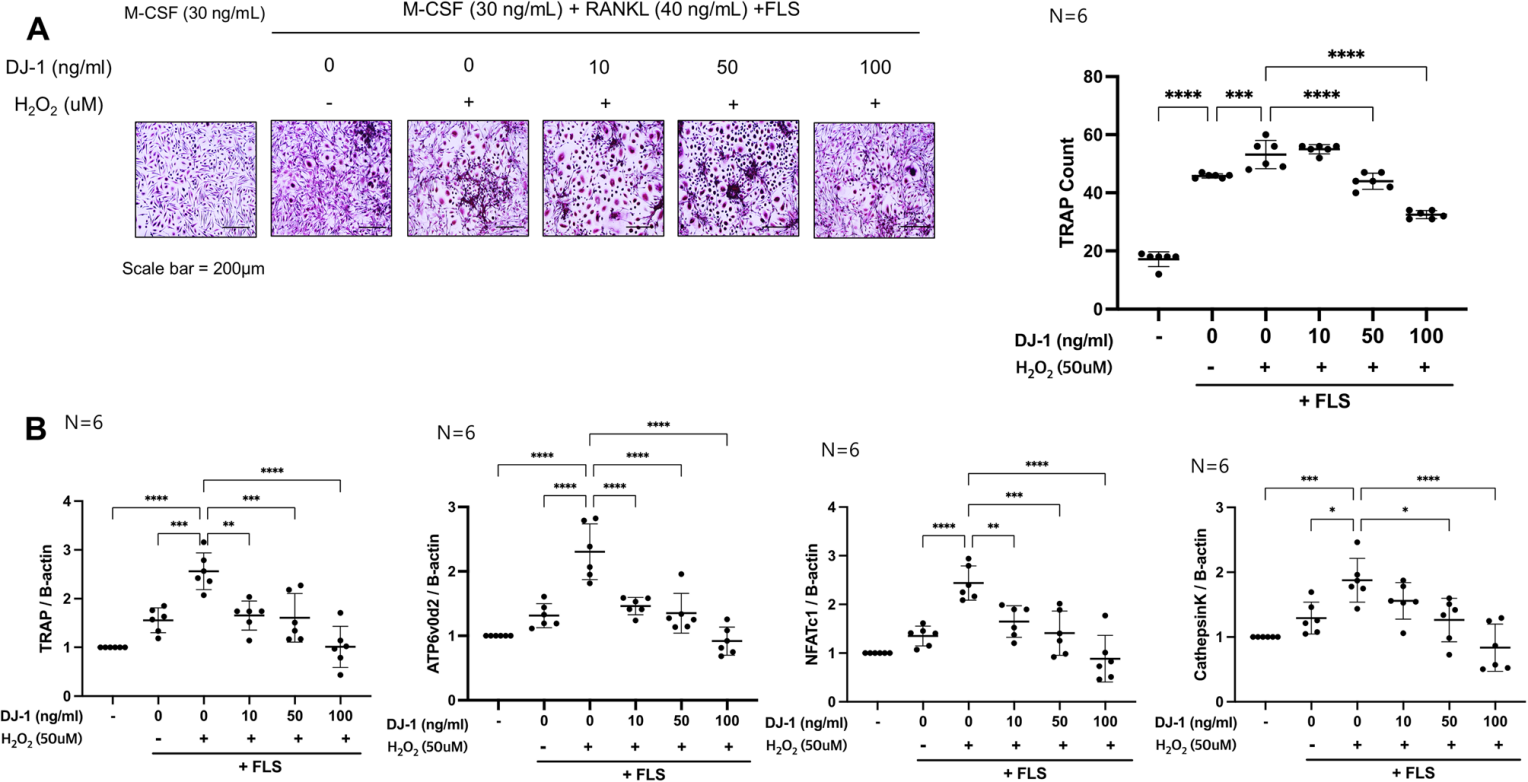
DJ-1-pretreated RA-FLSs suppress osteoclastogenesis. RA-FLS (2 × 10^3^ cells/well) were pretreated with H_2_O_2_ (50 μM) + DJ-1 (0, 10, 50, 100 ng/mL) for 3 days. Subsequently, RA-FLS were added to osteoclast culture plates containing M-CSF (25 ng/mL) pre-stimulated CD14^+^ monocytes (1 × 10^6^ cells/well). Then, RANKL (40 ng/mL) + M-CSF (30 ng/mL) were added and the medium was changed every 3 days. (**A**) Number of TRAP^+^ multinucleated cells. (**B**) Gene expression levels of osteoclast-related genes measured using RT-qPCR, and normalized to the expression levels of *ACTB*. *P < 0.05, **P < 0.01, ***P < 0.001, ****P < 0.0001. Scale bar = 200 μm.

RA-FLSs were cultured under 50 μM H_2_O_2_ with 0, 10, 50, and 100 ng/mL DJ-1 for 72 h, and added to osteoclast culture plates. TRAP^+^ cell counts were increased in the H_2_O_2_-stimulated RA-FLS-added group than in the H_2_O_2_ non-stimulated RA-FLS-added group, and TRAP^+^ cells decreased in a dose-dependent manner in DJ-1 10, 50, and 100 ng/mL pre-stimulated RA-FLS groups (Fig. 6A). In addition, the expression levels of osteoclast-related genes (*TRAP, ATP6v0d2, NAFTc1,* and *CTSK*) were suppressed in the DJ-1-pre-stimulated RA-FLS-added groups (Fig. 6B).

## Discussion

In the present study, we demonstrated that the serum and SF levels of DJ-1 and ROS were higher in RA patients than in OA patients. The addition of DJ-1 regulated Th17/Treg imbalance and suppressed the differentiation of RANKL-expressing CD4^+^ T cells and FLS. In addition, the levels of pro-inflammatory cytokines (TNF-α and IL-17A) and sRANKL production by Th cells were attenuated by DJ-1. ROS-stimulated FLS showed increased MMP-9 and sRANKL secretion, and DJ-1 reversed ROS-induced FLS activation. DJ-1-knockout mice showed higher arthritis scores and incidence rates than those of wild-type mice when an RA-like phenotype was induced by type II collagen immunization (collagen-induced mouse model)^30^; however, the mechanisms underlying the action of DJ-1 in RA progression were not assessed. In one study, RA-FLS stimulation by oxidative stress increased oxidation protein products and enhanced the gene expression levels of *TNF-α, IL-1β, MMPs*, and *VEGF* via the NADPH oxidase-dependent pathway^16^. Regarding T cell regulation by DJ-1, DJ-1 deficiency-induced Tregs showed a lower replicative and proliferative capacity^33^. In addition, DJ-1-knockout mice showed higher Th1 and Th17 responses and CD3^+^ T cell migration^38^, which implies a potential role of DJ-1 in T cell differentiation. Regulating the helper T cell subset imbalance is a possible treatment target for RA^39–41^; previous studies have found that upregulation of Th17 and downregulation of Treg (Th17/Treg imbalance) is evident in RA patients^19^. In the present study, DJ-1 administration regulated helper T cell subsets to suppress RA progression (downregulating Th17 and upregulating Treg) and diminished pro-inflammatory cytokine (IL-17A and TNF-α) secretion under Th17-polarizing conditions. Furthermore, TNF-α production was attenuated by DJ-1 administration in vitro following ROS stimulation of RA-FLS.

RANKL- and IL-17A-induced osteoclastogenesis were suppressed by DJ-1 treatment. Furthermore, ROS-activated RA-FLS promoted osteoclastogenesis, which was suppressed by adding DJ-1 to the RA-FLS pre-activation state. DJ-1 not only acts in the intracellular space, but can also be secreted^42^. Till date, little is known about the role of extracellular DJ-1. DJ-1 inhibits osteoclastogenesis by blocking intracellular ROS and subsequent TRAF6- and ITAM-mediated signaling pathways^30^. Osteoblasts are known as primary suppliers of RANKL, and the RANK-RANKL interaction is the primary activation signal for osteoclast differentiation and maturation^43^. In RA, other cells such as T cells and FLS can also express RANKL^23,24^, and these cells augment osteoclastogenesis in RA. One of the pivotal pro-inflammatory cytokines in RA pathogenesis is IL-17A, which can promote osteoclastogenesis without RANKL stimulation^25^. We demonstrated that osteoclastogenesis under RANKL and IL-17A stimulation was effectively suppressed by DJ-1 administration. In addition, H_2_O_2_ stimulated RA-FLS addition increased osteoclastogenesis, which was suppressed in DJ-1 co-stimulated RA-FLS-added osteoclastogenesis conditions. In addition, the expression of the osteoclast inducing factor, RANKL, was effectively suppressed in both in vitro PBMC culture (under Th17-polarizing condition) and RA-FLS culture (under ROS stimulation) by DJ-1 administration. These results differed from those of a previous study^30^ with respect to the method (DJ-1-knockout versus extracellular addition of DJ-1) used and the osteoclast stimulation conditions (IL-17A stimulation). Furthermore, co-culture of osteoclasts and RA-FLS reinforces the idea that DJ-1 exerts both a direct and indirect suppressive role on osteoclastogenesis via regulation of FLS. The present results suggest the potential therapeutic potential of DJ-1 in RA, and the beneficial effects of DJ-1 could also be exerted by external administration.

Rapid and vigorous proliferation of the synovium and pannus formation are the cornerstone pathologic processes of RA^1^, and pannus formation requires higher oxygen delivery. Expression of angiogenetic factors, such as VEGF, is increased in the serum and SF of RA patients^44^, and these angiogenic factors promote neo-angiogenesis of the pannus^45^. In addition, the oxidative stress marker of SF positively correlated with the DAS28 of RA patients^46^. This implies that decreasing oxidative stress via an ROS scavenger such as DJ-1 may be a potential therapeutic target in RA treatment. VEGF can induce osteoclastogenesis in RA by dual action: (1) inducing RANKL expression in RA-FLS, and (2) directly inducing osteoclast differentiation in osteoclast precursor cells^47^. Among the various MMPs, MMP-9 promotes RA-FLS survival and cartilage destruction^48^. Furthermore, in a mouse model of RA, *MMP-9*-knockout mice showed less severe arthritis than wild-type mice^49^, indicating the pivotal role of MMP-9 in RA pathogenesis. In the present study, ROS stimulation increased VEGF and MMP-9 production in RA-FLS, and DJ-1 suppressed ROS-induced VEGF and MMP-9 production in RA-FLS.

## Conclusions

In conclusion, we demonstrated the therapeutic potential of DJ-1 in RA pathogenesis. DJ-1 controls the pathologic Th cell subset (Th17, RANKL^+^CD4^+^ T cells), induces Treg differentiation, and inhibits pro-inflammatory cytokine production. ROS-stimulated RA-FLS exhibited an attenuated inflammatory response and RANKL/MMP-9/VEGF expression following DJ-1 treatment. Finally, osteoclastogenesis was attenuated by DJ-1 administration under RANKL/IL-17A stimulated condition. In addition, DJ-1 addition attenuated osteoclastogenesis induced by ROS-stimulated RA-FLS. These findings support the therapeutic potential of DJ-1 exerted via several molecular pathways in RA pathogenesis.

## Supplementary Information

Supplementary Figure 1.

Supplementary Information.
