# Supplementary figures and images for: DJ-1 controls T cell differentiation and osteoclastogenesis in rheumatoid arthritis
Source: Sci Rep. 2022 Jul 27;12:12767. doi: 10.1038/s41598-022-16285-1 (PMC9329329; doi:10.1038/s41598-022-16285-1)

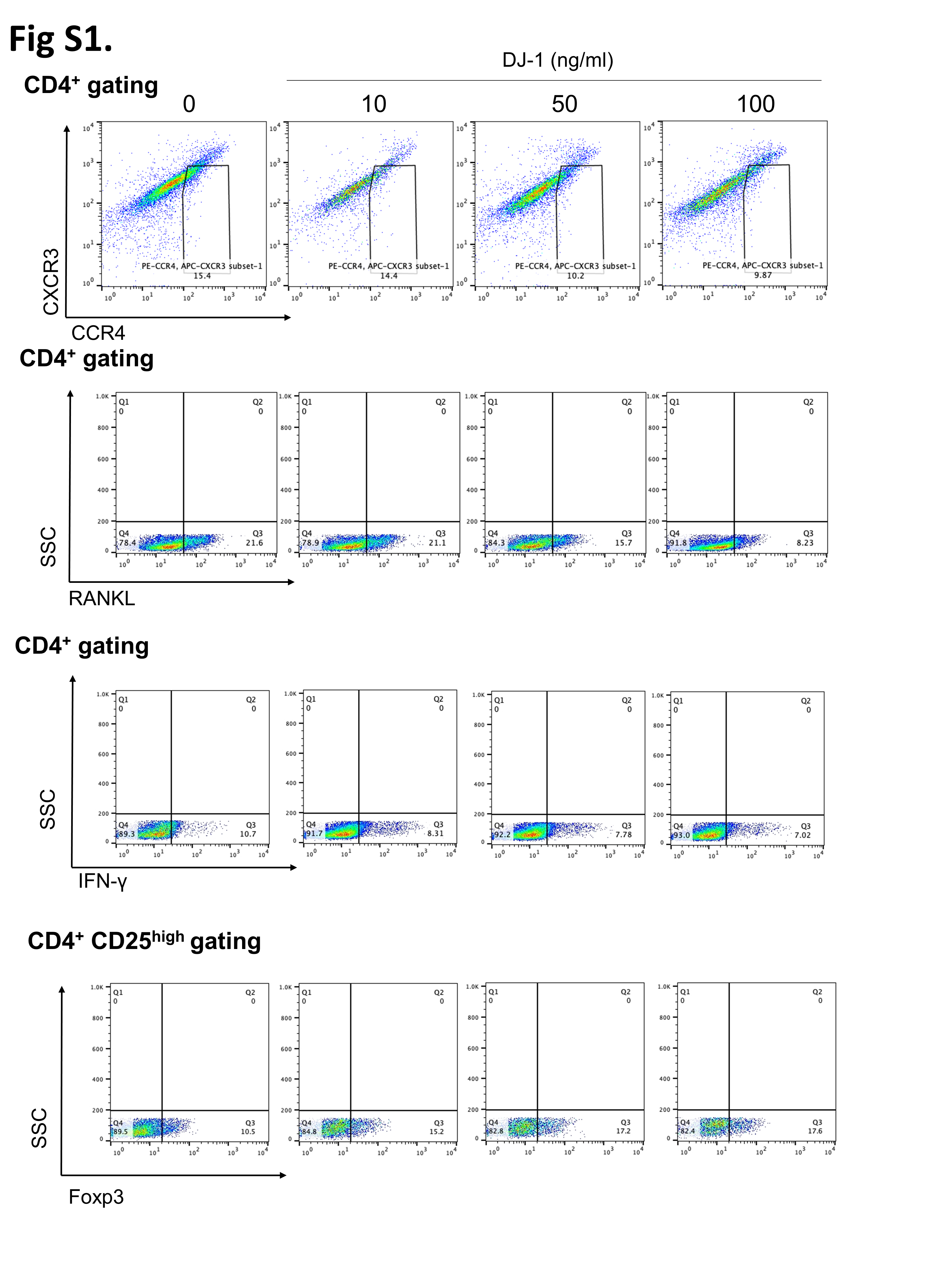

Supplement: Supplementary file 1 — Supplementary Figure 1. [file 41598_2022_16285_MOESM1_ESM.tif]
