# Supplementary material for: DJ-1 controls T cell differentiation and osteoclastogenesis in rheumatoid arthritis
Source: Sci Rep. 2022 Jul 27;12:12767. doi: 10.1038/s41598-022-16285-1 (PMC9329329; doi:10.1038/s41598-022-16285-1)
Supplement: Supplementary file 2 — Supplementary Information. [file 41598_2022_16285_MOESM2_ESM.docx]

**Supplementary Table 1.** Baseline characteristics of RA patients

|  | RA |
| --- | --- |
|  | (N=10) |
| Age (years) | 62.5 [50.0;70.0] |
| Female sex (N, %) | 7 (70.0%) |
| Disease duration (years) | 2.5 [ 1.0; 5.0] |
| Rheumatoid factor positive (N, %) | 7 (70.0%) |
| Anti-citrullinated protein Ab positive (N, %) | 6 (60.0%) |
| ESR (mm/hr) | 30.0 [ 4.0;52.0] |
| CRP (mg/dL) | 0.2 [ 0.1; 1.1] |
| DAS28-ESR | 3.4 [ 2.5; 4.7] |
| Medication |  |
| Methotrexate (N, %) | 9 (90.0%) |
| Sulfasalazine (N, %) | 4 (40.0%) |
| Hydroxychloroquine (N, %) | 5 (50.0%) |
| Leflunomide (N, %) | 1 (10.0%) |
| Glucocorticoid | 5 (50%) |
| b/tsDMARDs | 0 |

RA: rheumatoid arthritis; ESR: erythrocyte sedimentation rate; CRP: C-reactive protein; DAS28: disease activity score-28 joints; b/ts DMARDs: biologic / targeted synthetic disease-modifying antirheumatic drugs

**Supplementary figure 1.** Flow cytometry gate strategies for CD4^+^ CCR4^+^ CCR6^+^ CXCR3^-^ T cells, CD4^+^ RANKL^+^ T cells, CD4^+^ IFN-γ^+^ T cells, and CD4^+^ CD25^high^ Foxp3^+^ T cells.
